# Signaling through alternative Integrated Stress Response pathways compensates for GCN2 loss in a mouse model of soft tissue sarcoma

**DOI:** 10.1038/srep11781

**Published:** 2015-06-30

**Authors:** Stacey L. Lehman, Sandra Ryeom, Constantinos Koumenis

**Affiliations:** 1 Deparment of Radiation Oncology, Perelman School of Medicine at the University of Pennsylvania, Philadelphia, PA, USA 19104; 2 Department of Cancer Biology, Perelman School of Medicine at the University of Pennsylvania, Philadelphia, PA, USA 19104

## Abstract

The tumor microenvironment is characterized by deficiencies in oxygen and nutrients, such as glucose and amino acids. Activation of the GCN2 arm of the Integrated Stress Response (ISR) in response to amino acid deprivation is one mechanism by which tumor cells cope with nutrient stress. GCN2 phosphorylates the alpha subunit of the eukaryotic translation initiation factor eIF2, leading to global downregulation of translation to conserve amino acids and initiation of a transcriptional program through ATF4 to promote recovery from nutrient deprivation. Loss of GCN2 results in decreased tumor cell survival *in vitro* under amino acid deprivation and attenuated tumor growth in xenograft tumor models. However, it is not known what effects GCN2 loss has on the growth of autochthonous tumors that arise in their native microenvironment. Here, we demonstrate in a genetically engineered mouse model of soft tissue sarcoma that loss of GCN2 has no effect on tumor growth or animal survival. The sarcomas displayed compensatory activation of PERK or phospho-eIF2α independent upregulation of ATF4 in order to maintain ISR signaling, indicating that this pathway is critical for tumorigenesis. These results have important implications for the development and testing of small molecule inhibitors of ISR kinases as cancer therapeutics.

Cells have evolved a variety of pathways to combat both intrinsic and extrinsic stressors. One such pathway is the Integrated Stress Response (ISR), which consists of four kinases that converge on phosphorylation of the eukaryotic translation initiation factor eIF2α in response to a diverse array of stimuli. These kinases are HRI, which responds to heme deprivation, PKR, which responds to double-stranded RNA, PERK, which responds to accumulation of unfolded proteins, and GCN2, which responds to amino acid deprivation^1^. ISR activation, and the resulting phosphorylation of eIF2α, has two major consequences. The first of these is global downregulation of translation^2^. The second is the translational upregulation of specific transcripts that contain upstream open reading frames (uORFs) in their 5’ untranslated region (UTR). The best characterized of these is the transcription factor ATF4^3^,^4^,^5^. Translational upregulation of ATF4 leads to activation of a transcriptional program of genes that regulates a variety of processes, including amino acid transport and synthesis, redox balance, and autophagy^6^,^7^. Both of these outcomes help to promote cell survival under conditions of stress, but prolonged or severe activation of the ISR can lead to apoptosis^1^.

Many of the stressors that activate the ISR are encountered in the tumor microenvironment. Tumor vasculature is often poorly constructed, resulting in leaky, tortuous vessels that cannot deliver nutrients efficiently to tumor cells. The high metabolic rate of tumor cells also leads to rapid consumption of nutrients that are available to the cells. This results in oxygen, glucose, and amino acid deprivation in areas of the tumor^8^. Hypoxia and glucose deprivation both interfere with proper protein folding, resulting in endoplasmic reticulum (ER) stress and PERK activation^9^,^10^, while amino acid deprivation activates GCN2^11^,^12^. The cell autonomous stress of oncogene activation through c-Myc places high demands on protein synthesis and can also activate PERK^13^.

Previous research from many groups, including our own, has shown that ISR activation promotes tumor cell survival. *In vitro*, cells lacking PERK exhibit decreased clonogenic survival after exposure to hypoxia^14^. PERK^−/−^ cells with activated c-Myc also have greatly reduced clonogenic survival^13^. *In vivo*, PERK^−/−^ cells injected into the flanks of nude mice form much smaller tumors than their wildtype counterparts^14^,^15^. Additionally, hypoxic and apoptotic areas of PERK null tumors overlap, indicating that PERK supports tumor cell survival under conditions of hypoxia in the microenvironment^14^. Similarly, cells lacking GCN2 undergo apoptosis when grown in media lacking an amino acid. Loss of GCN2 in xenograft models of cancer greatly inhibits or even prevents tumor growth^16^.

While these results are promising, it is necessary to test the role of ISR kinases in genetically engineered mouse models (GEMMs) of cancer. The majority of conditions known to activate the ISR in tumor cells, such as hypoxia and low nutrient levels, are non-cell autonomous and dictated by the tumor microenvironment. Cells grown subcutaneously in mice as xenografts are not exposed to physiological levels of microenvironmental stress. Many important components of the tumor microenvironment, such as stromal cells, immune cells, and blood vessels, are altered or absent in xenograft models^17^ . GEMMs provide the benefit of modeling the growth of tumors in their native microenvironment, which allows for a more accurate assessment of the contribution of these kinases to tumor growth.

The ISR kinase PERK has been studied in GEMMs. PERK^fl/fl^;MMTV-cre mice crossed to MMTV-neu mice resulted in the specific deletion of PERK in the mammary glands of tumor-prone MMTV-neu mice. Results from this study showed that loss of PERK in mammary carcinomas slowed tumor growth and reduced the number of metastases to the lungs^18^. In a second model, mice expressing SV40 large T antigen specifically in the beta cells of the pancreas were crossed to PERK^−/−^ mice. Loss of PERK resulted in the formation of fewer and smaller insulinomas in mice^19^. In general, the results of PERK loss in GEMMs recapitulate the observations made in xenograft models.

Although the role of PERK in tumorigenesis has been characterized in both xenograft and genetically engineered mouse models of cancer, GCN2 has yet to be studied in a GEMM. Our previous studies in the HT1080 human sarcoma cell line revealed that knockdown of GCN2 completely blocked tumor growth in a xenograft model^16^. Here, we expand our studies to GCN2 null mice crossed to a genetic model of soft tissue sarcoma developed by Kirsch and colleagues^20^. Surprisingly, in autochthonous tumors, loss of GCN2 did not affect tumor initiation or progression and did not increase the lifespan of tumor-bearing mice. Analysis of the tumors at the molecular level revealed several mechanisms of compensation for GCN2 loss. Mice on a C57BL6 background exhibited increased signaling through PERK to maintain levels of phosphorylated eIF2α in tumors, while mixed background mice upregulated ATF4 independently of eIF2α phosphorylation to maintain ISR signaling. Overall, this study reveals the importance of ISR signaling in tumorigenesis and defines mechanisms by which pleiomorphic sarcomas, and potentially other tumor types as well, can become resistant to ISR inhibition. As small molecule inhibitors of the ISR kinases are currently under development, this study has clinical relevance to their testing in animal models and insight into ways tumor cells may circumvent their effects.

## Results

**Figure 1:**
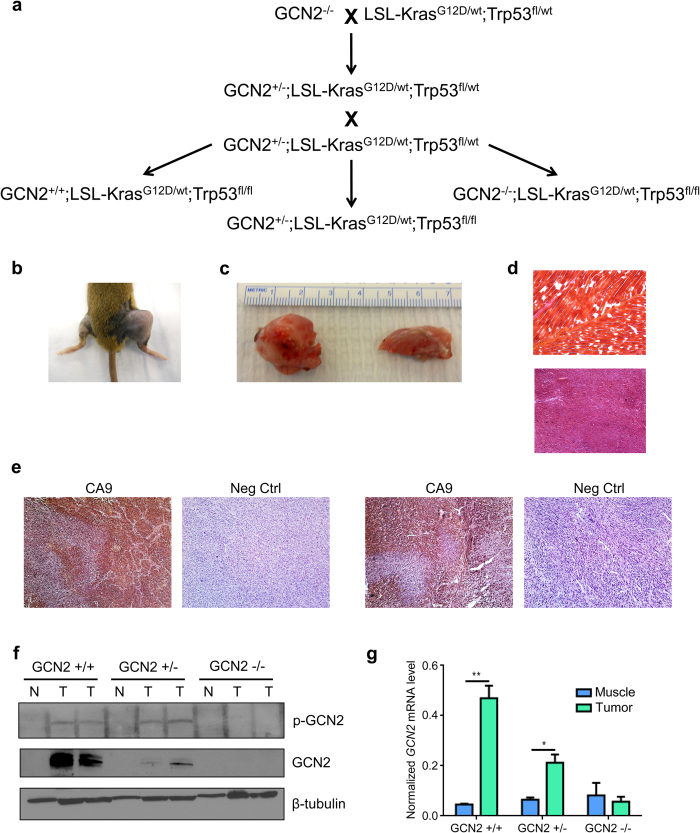
Development of a genetically engineered mouse model to study the role of GCN2 in sarcomagenesis. (**a**) Breeding scheme to generate GCN2^+/+^, GCN2^+/−^, and GCN2^−/−^ mice on an LSL-Kras^G12D/wt^;p53^fl/fl^ background. (**b**) Gross morphology of sarcomas compared to normal leg. The right leg was injected with Ad-cre, while the left leg served as a normal tissue control. (**c**) Gross morphology of sarcomas compared to normal leg. The size of a typical sarcoma (left) is compared to the size of the normal leg (right) from the same mouse. (**d**) Typical histology of normal muscle (top) and soft tissue sarcoma (bottom) stained with hematoxylin and eosin. Magnification is 40X. Normal muscle and tumor shown are from the same animal. (**e**) Typical immunohistochemistry for CA9 in two sarcomas, along with the no primary antibody control. Tissue sections were counterstained with hematoxylin. Magnification is 100X. (**f**) Western blot analysis of phosphorylated and total GCN2 in homogenized normal muscle tissue (N) and soft tissue sarcomas (T). β-tubulin was used as a loading control. (**g**) qPCR analysis of *GCN2* levels in sarcoma and muscle tissue. *GCN2* levels were normalized to the geometric mean of the reference genes β-actin and 18S rRNA. Data are represented as the average value for each genotype ± standard error of the mean; *p < 0.05, **p < 0.01.

To study the role of GCN2 in tumorigenesis, we crossed GCN2^−/−^ mice to LSL-Kras^G12D/wt^;p53^fl/fl^ mice to obtain GCN2 wildtype, heterozygous, and null mice on an LSL-Kras^G12D/wt^;p53^fl/fl^ background (Fig. 1a). Sarcoma formation was induced in these mice by injection of adenovirus expressing cre recombinase (Ad-cre) into the right leg muscle. This resulted in the formation of soft tissue sarcomas in nearly 100% of mice. Grossly, the sarcomas grew rapidly and essentially took over the normal muscle tissue in the leg (Fig. 1b,c). At the microscopic level, the tumors were highly dedifferentiated with complete loss of normal tissue architecture (Fig. 1d). To determine if the tumors were undergoing oxygen and nutrient deprivation, hypoxia levels were assessed by performing immunohistochemistry for the hypoxia marker carbonic anhydrase 9 (CA9). Tumors demonstrated extensive areas of hypoxia (Fig. 1e).

To determine if the GCN2 arm of the ISR was activated in the sarcomas, we homogenized tumors and normal muscle from GCN2^+/+^, GCN2^+/−^, and GCN2^−/−^ mice and immunoblotted for total and phosphorylated GCN2. As we previously observed in human patient samples^16^, GCN2 was overexpressed in tumor tissue as compared to normal tissue in wildtype and heterozygous mice. We also detected phosphorylation of GCN2 in the wildtype and heterozygous mice, indicating that GCN2 was activated in the tumors (Fig. 1f). Since GCN2 in normal muscle tissue could not be detected by western blot, its overexpression at the mRNA level was confirmed by qPCR (Fig. 1g).

**Figure 2:**
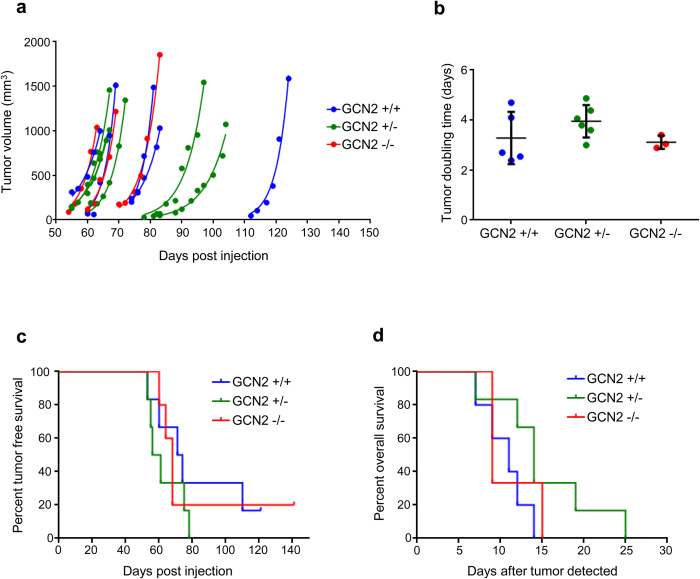
GCN2 does not affect tumor growth or survival of sarcoma-bearing mice on a mixed background. (**a**) Measurements of tumor volume over time in GCN2^+/+^, GCN2^+/−^, and GCN2^−/−^ mixed background mice. The line through the set of measurements for each mouse represents the best fit exponential growth equation of the tumor. (**b**) Tumor doubling time was calculated from the best fit exponential growth equations shown in (**a**). The average doubling time ± standard deviation is depicted for each genotype. Results were not statistically significant. (**c**) Kaplan-Meier curves depicting the time from Ad-cre injection to tumor formation for each GCN2 genotype. Results were not statistically significant. (**d**) Kaplan-Meier curves depicting the time from tumor detection to euthanasia for each GCN2 genotype. The major euthanasia criterion was a tumor volume exceeding 1000 mm^3^. Results were not statistically significant.

Tumor volume was measured in the mice over time. As shown in Fig. 2a, palpable tumors formed in most mice between 50 to 80 days post-injection. Tumor measurements were fit to exponential growth curves, and doubling times were calculated from the growth curves to determine the tumor growth rates. Loss of GCN2 did not affect tumor growth. Regardless of the GCN2 status of the mice, all tumors had a doubling time of three to four days, and the differences among the three genotypes were not statistically significant (Fig. 2b).

Kaplan-Meier curves were generated for both time to tumor formation after Ad-cre injection and time to death after tumor detection. There were no statistically significant differences in survival among the three genotypes, indicating that GCN2 did not affect tumor initiation or tumor progression in this model (Fig. 2c,d). To ensure that using both male and female mice did not obscure the effects of GCN2, we also generated survival curves based on the sex of the mice. These data show that tumor initiation and progression were identical in males and females (Supplementary Fig. S1).

The GCN2^−/−^ mice used in this study were on a C57BL6 background, while the LSL-Kras^G12D/wt^;p53^fl/fl^ mice were on a mixed background. To determine if genetic heterogeneity of the mixed background mice masked the effects of GCN2, we obtained C57BL6 LSL-Kras^G12D/wt^;p53^fl/fl^ mice to perform the crosses again to carry out the experiments on a uniform genetic background. While tumors initiated in the C57BL6 mice within a much narrower window (40 to 50 days for most mice), loss of GCN2 still had no effect on tumor growth rate, tumor free survival, or overall survival of the mice (Supplementary Fig. S2). We also confirmed that there was no difference in survival between C57BL6 males and females (Supplementary Fig. S3).

**Figure 3:**
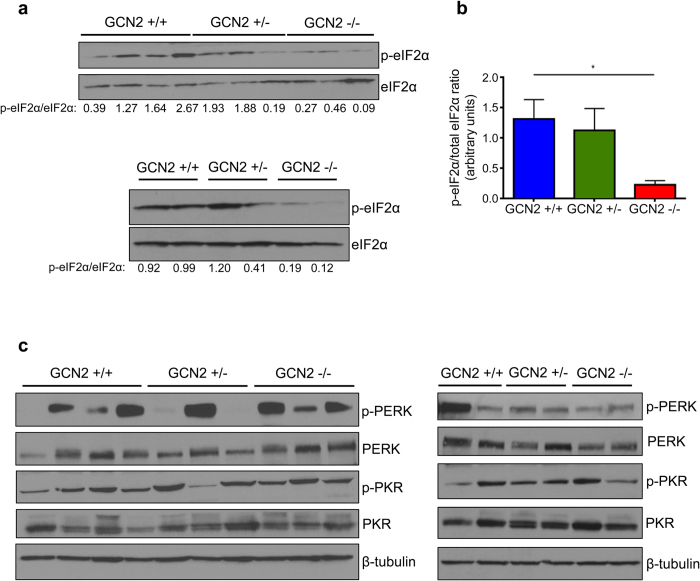
Loss of GCN2 reduces eIF2α phosphorylation in mixed background sarcomas. (**a**) Western blot analysis on two independent samples of tumor homogenates for levels of phosphorylated and total eIF2α in mixed background tumors. The ratio of phosphorylated to total eIF2α is displayed below each blot. (**b**) Graphical representation of the phosphorylated to total eIF2α ratios calculated from the blots in (**a**). Data are represented as the average value for each genotype ± standard error of the mean; *p < 0.05. (**c**) Western blot analysis on two independent samples of tumor homogenates for levels of phosphorylated and total PERK and phosphorylated and total PKR in mixed background tumors. β-tubulin was used as a loading control.

Next, we wanted to determine if loss of GCN2 affected downstream signaling through the ISR in tumor tissue. Western blot analysis of tumor lysates revealed that loss of GCN2 did reduce levels of eIF2α phosphorylation in mixed background mice. We repeated the analysis on a small, independent sample of tumors and found similar results (Fig. 3a). Quantification of the p-eIF2α to total eIF2α ratio showed that GCN2^−/−^ mixed background sarcomas had a statistically significant decrease in eIF2α phosphorylation (Fig. 3b). We also examined signaling through other branches of the ISR by measuring PERK and PKR phosphorylation in the tumors. PERK phosphorylation was detected in almost all tumors to varying degrees, while PKR was phosphorylated rather uniformly in all tumors (Fig. 3c). These data indicated that all relevant branches of the ISR are activated in this sarcoma model and that PERK and PKR are responsible for the residual eIF2α phosphorylation detected in GCN2^−/−^ tumors. An analysis of normal muscle tissue indicated that, similar to GCN2, the sarcomas had overexpressed and activated PERK and PKR. Total eIF2α and phospho-eIF2α were also increased in tumors as compared to normal tissue controls (Supplementary Fig. S4a). Staining of the membrane with Ponceau revealed that, overall, normal muscle tissue and sarcoma tissue have very different patterns of protein expression (Supplementary Fig. S4b).

**Figure 4:**
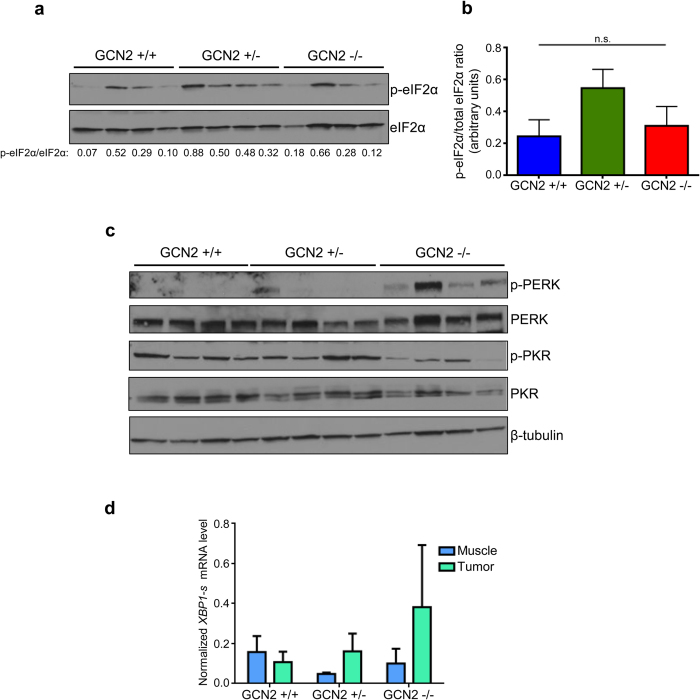
Loss of GCN2 does not reduce eIF2α phosphorylation in C57BL6 sarcomas, potentially due to compensation by PERK. (**a**) Western blot of tumor homogenates for levels of phosphorylated and total eIF2α in C57BL6 tumors. The ratio of phosphorylated to total eIF2α is displayed below each blot. (**b**) Graphical representation of the phosphorylated to total eIF2α ratios calculated from the blots in (**a**). Data are represented as the average value for each genotype ± standard error of the mean. Results are not statistically significant. (**c**) Western blot analysis of tumor homogenates for levels of phosphorylated and total PERK and phosphorylated and total PKR in C57BL6 tumors. β-tubulin was used as a loading control. (**d**) qPCR analysis of spliced *XBP1* levels in sarcoma and muscle tissue. Spliced *XBP1* levels were normalized to the geometric mean of the reference genes β-actin and 18S rRNA. Data are represented as the average value for each genotype ± standard error of the mean.

We repeated our study of ISR signaling in the C57BL6 tumors. Interestingly, the GCN2 status of these mice did not affect levels of eIF2α phosphorylation in the sarcomas (Fig. 4a). There was no statistically significant difference in the p-eIF2α to total eIF2α ratio in GCN2^−/−^ mice as compared to the other genotypes (Fig. 4b). As we observed in the mixed background tumors, PKR was phosphorylated in all C57BL6 tumors. However, substantially increased PERK phosphorylation was detected almost exclusively in GCN2^−/−^ tumors (Fig. 4c). This suggests that in the C57BL6 tumors, PERK compensates for loss of GCN2, resulting in maintenance of eIF2α phosphorylation levels. One potential explanation for the nearly exclusive activation of PERK in GCN2^−/−^ tumors is that GCN2 is the primary ISR kinase responsible for eIF2α phosphorylation in the tumors. When GCN2 is not present, tumor cells lose their ability to suppress translation under stress, causing them to deplete their ATP supplies. The drop in ATP levels would affect chaperone activity, resulting in the accumulation of unfolded proteins and PERK activation. This situation is not unique to cancer, as in Alzheimer’s disease, another pathological condition that activates the ISR, knockout of GCN2 also results in compensatory activation of PERK in the brains of affected animals, leading to no improvements in memory decline^21^. To determine if other arms of the Unfolded Protein Response (UPR) were activated in addition to PERK, we performed qPCR on RNA isolated from normal muscle and tumor for spliced XBP1. Enhanced XBP1 splicing in tumors was observed in GCN2^−/−^ mice, suggesting that loss of GCN2 sensitizes cells to ER stress (Fig. 4d).

**Figure 5:**
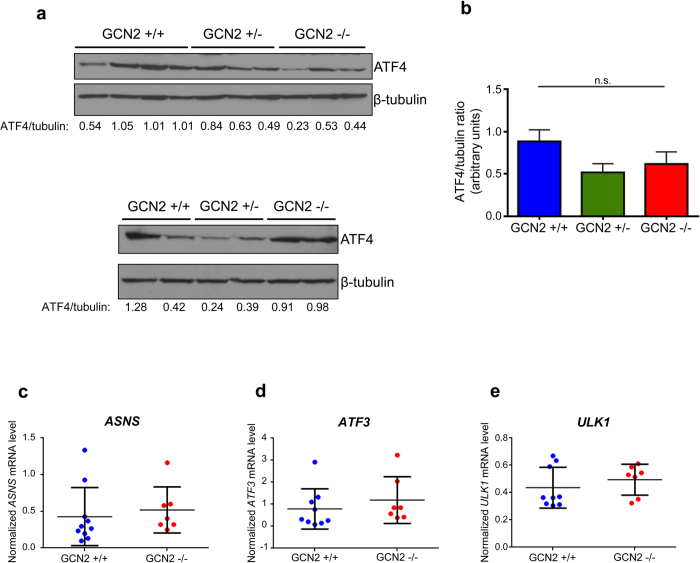
GCN2^−/−^ mixed background sarcomas do not express lower levels of ATF4, in spite of reduced eIF2α phosphorylation. (**a**) Western blot analysis on two independent samples of tumor homogenates for levels of ATF4 in mixed background tumors. β-tubulin was used as a loading control. The ratio of ATF4 to β-tubulin is displayed below each blot. (**b**) Graphical representation of the ATF4 to β-tubulin ratios calculated from the blots in (**a**). Data are represented as the average value for each genotype ± standard deviation. Results are not statistically significant. (**c**) qPCR analysis of *ASNS* levels in GCN2^+/+^ and GCN2^−/−^ sarcomas. *ASNS* levels were normalized to the geometric mean of the reference genes β-tubulin, β-actin, and 18S rRNA. Data are represented as the average value for each genotype ± standard deviation. Results are not statistically significant. (**d**) qPCR analysis of *ATF3* levels in GCN2^+/+^ and GCN2^−/−^ sarcomas. *ATF3* levels were normalized to the geometric mean of the reference genes β-tubulin, β-actin, and 18S rRNA. Data are represented as the average value for each genotype ± standard deviation. Results are not statistically significant. (**e**) qPCR analysis of *ULK1* levels in GCN2^+/+^ and GCN2^−/−^ sarcomas. *ULK1* levels were normalized to the geometric mean of the reference genes β-tubulin, β-actin, and 18S rRNA. Data are represented as the average value for each genotype ± standard deviation. Results are not statistically significant.

Next, we wanted to examine ISR signaling downstream of eIF2α phosphorylation by measuring ATF4 levels in tumors. As expected from the similar levels of eIF2α phosphorylation, GCN2 status did not affect ATF4 protein levels in C57BL6 tumors (Supplementary Fig. S5). However, in the mixed background GCN2^−/−^ tumors, in which there was a significant decrease in eIF2α phosphorylation, ATF4 protein levels were minimally affected (Fig. 5a). While there was a small decrease in ATF4 in both GCN2^−/−^ and GCN2^+/−^ tumors, it was not statistically significant (Fig. 5b). To further confirm that ATF4 was expressed similarly regardless of GCN2 status, we measured mRNA levels of the ATF4 target genes ASNS, ATF3, and ULK1 by qPCR. All three genes were expressed at the same level in GCN2^+/+^ and GCN2^−/−^ tumors (Fig. 5c–e). This suggested to us that the mixed background tumors compensated for GCN2 loss by upregulating ATF4 independently of eIF2α phosphorylation levels.

**Figure 6:**
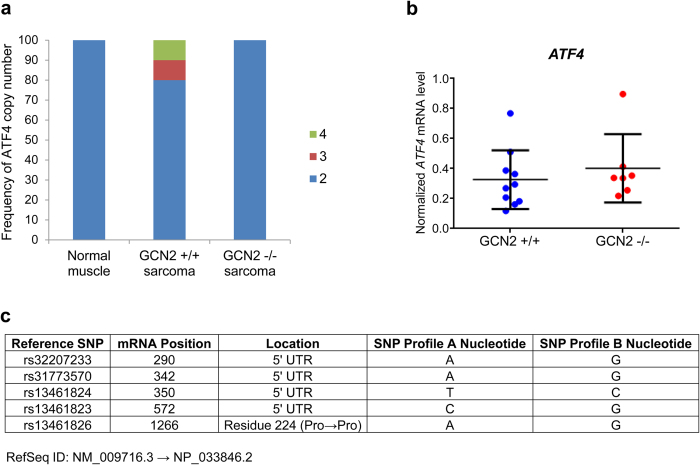
Maintenance of ATF4 protein expression in mixed background GCN2^−/−^ sarcomas likely occurs *in trans*. (**a**) Copy number analysis of ATF4 in normal muscle tissue, GCN2^+/+^ sarcomas, and GCN2^−/−^ sarcomas in mixed background mice. Copy number increases were detected in two GCN2^+/+^ sarcomas (normal muscle n = 3, GCN2^+/+^ sarcoma n = 10, GCN2^−/−^ sarcoma n = 7). (**b**) qPCR analysis of *ATF4* levels in GCN2^+/+^ and GCN2^−/−^ sarcomas. *ATF4* levels were normalized to the geometric mean of the reference genes β-tubulin, β-actin, and 18S rRNA. Data are represented as the average value for each genotype ± standard deviation. Results are not statistically significant. (**c**) ATF4 SNPs detected in mixed background sarcomas. Two variations of ATF4 alleles were found in the mice, with five SNPs clustering in distinct patterns. These are designated as SNP Profile A and SNP Profile B. The nucleotide position of each SNP in the mRNA is indicated, along with the region of the mRNA in which the SNP is located.

One mechanism by which GCN2^−/−^ tumors could upregulate ATF4 is by increasing levels of *ATF4* transcript in order to compensate for lower levels of eIF2α phosphorylation. This could be accomplished directly through transcriptional upregulation or indirectly through an increase in ATF4 copy number. We first measured ATF4 copy number in GCN2^+/+^ and GCN2^−/−^ sarcomas with a TaqMan copy number assay. Normal muscle tissue was used as a copy number control. ATF4 amplification was observed in two GCN2^+/+^ tumors, but no GCN2^−/−^ tumors (Fig. 6a). qPCR analysis of *ATF4* mRNA in GCN2^+/+^ and GCN2^−/−^ tumors demonstrated equal levels of transcript in both genotypes (Fig. 6b). Thus, it appears that GCN2^−/−^ sarcomas do not directly or indirectly upregulate *ATF4* transcript levels.

A second mechanism by which GCN2^−/−^ tumors could upregulate ATF4 is through mutations in the protein that enhance its expression levels. For example, mutations of the 5’ uORFs could uncouple ATF4 expression from eIF2α phosphorylation, and mutations in the β-TrCP recognition motif or the oxygen dependent degradation (ODD) domain could enhance ATF4 protein stability. To assess this possibility, we sequenced the three exons of ATF4 in both GCN2^+/+^ and GCN2^−/−^ tumors. *De novo* mutations were not detected in either GCN2^+/+^ or GCN2^−/−^ tumors. However, there were differences in previously known ATF4 SNPs. Five SNPs clustered into two distinct groups, which we designated SNP Profile A and B (Fig. 6c and Supplementary Fig. S6). The majority of mice were heterozygous at all five loci, although we did find both GCN2^+/+^ and GCN2^−/−^ mice homozygous for either SNP Profile A or B. Four of the five SNPs are located in the 5’ UTR of ATF4, but they do not interfere with the 5’ uORFs. The remaining SNP in the coding region results in a synonymous codon change. Thus, it does not appear that these SNPs would affect ATF4 expression levels. The lack of substantial differences in ATF4 copy number, transcript levels, and coding sequence between GCN2^+/+^ and GCN2^−/−^ mixed background sarcomas suggests that another factor is regulating ATF4 protein expression *in trans* to maintain ATF4 levels in GCN2^−/−^ tumors. Although the mechanism is unclear, this could be achieved by downregulation of proteins responsible for ATF4 degradation, such as β-TrCP^22^ or PHD3^23^, or upreguation of proteins that stabilize ATF4, such as p300^24^. Phosphorylation of ATF4 also regulates its stability^22^, so changes in this post-translational modification could also impact ATF4 levels in GCN2^−/−^ tumors.

**Figure 7:**
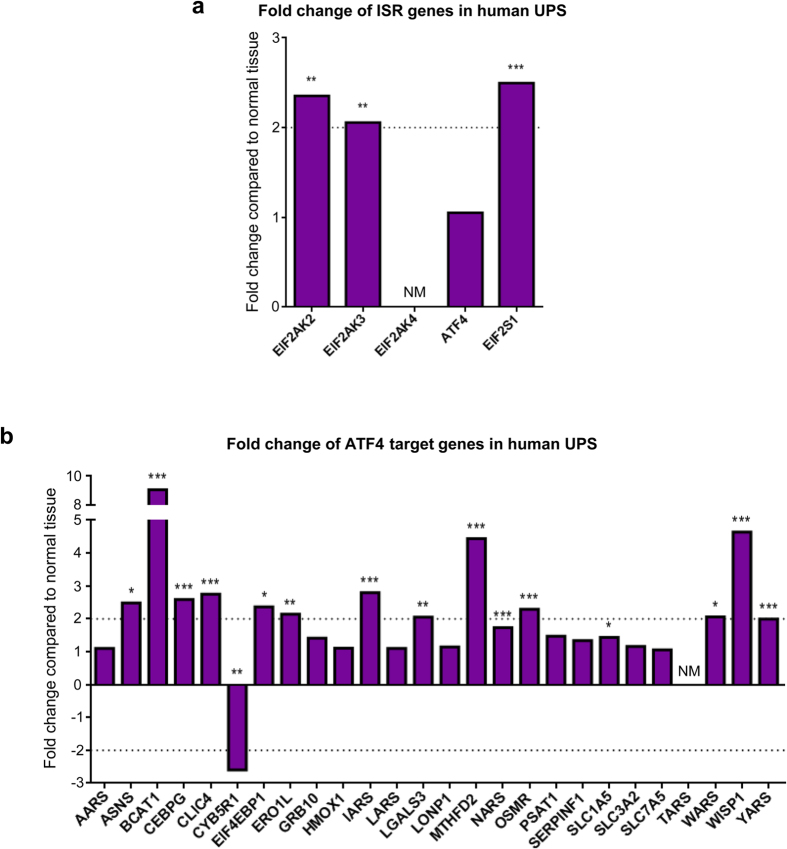
Many genes involved in ISR signaling are overexpressed in human undifferentiated pleomorphic sarcoma. (**a**) Analysis of overexpression of ISR-related genes in human undifferentiated pleomorphic sarcoma using the Detwiller dataset in Oncomine. Data are represented as fold change over normal tissue control; **p < 0.01, ***p < 0.001, NM = not measured. (**b**) Analysis of over- and underexpression of ATF4 target genes in human undifferentiated pleomorphic sarcoma using the Detwiller dataset in Oncomine. Data are represented as fold change over normal tissue control; *p < 0.05, **p < 0.01, ***p < 0.001, NM = not measured.

Our results in this mouse model demonstrate that ISR signaling is an important component of sarcoma biology. To determine if there is evidence of enhanced ISR signaling in human sarcomas, we used the online database Oncomine (www.oncomine.org, Compendia Bioscience, Ann Arbor, MI) to compare the expression of ISR-related genes in sarcoma samples and normal tissue controls using the Detwiller sarcoma dataset^25^. We chose to focus on undifferentiated pleomorphic sarcoma (UPS), also known as malignant fibrous histiocytoma (MFH), since the LSL-Kras^G12D/wt^;p53^fl/fl^ model most closely resembles this sarcoma subtype at the genetic level^26^. Both PKR (EIF2AK2) and PERK (EIF2AK3) were overexpressed in UPS samples, along with eIF2α (EIF2S1). Unfortunately, GCN2 (EIF2AK4) expression was not measured in this study (Fig. 7a).

ATF4 mRNA levels were the same in normal tissue and tumor tissue. However, this is not surprising since ATF4 is primarily regulated at the translational level. As an indirect way to examine ATF4 expression, we analyzed a number of ATF4 transcriptional targets in the same dataset. To assemble an unbiased list of ATF4 targets, we utilized published microarray data to identify genes whose expression was reduced by at least two-fold in ATF4^−/−^ cells treated with the ER stress inducing agent tunicamycin as compared to tunicamycin-treated ATF4^+/+^ cells^6^. To ensure that only direct ATF4 transcriptional targets were analyzed, we used a published ChIP-seq study to exclude genes whose promoters were not bound by ATF4 during tunicamycin treatment^27^. This resulted in a list of 26 ATF4 targets, which included genes such as amino acyl tRNA synthetases, amino acid transporters, and enzymes regulating redox balance. Of the 26 ATF4 target genes, 15 were significantly overexpressed in human UPS samples as compared to normal tissue. All but two of these genes were expressed in sarcomas at levels at least two-fold higher than normal tissue. Only one gene of the 26 was significantly underexpressed, and one gene from the list was not measured in this study. The remaining genes did not show evidence of differential expression (Fig. 7b). This analysis reveals that many ATF4 target genes are overexpressed in human patient samples, indicating that ISR activation in sarcomas is a clinically relevant phenomenon and not just restricted to mouse models.

## Discussion

This study demonstrates the role of GCN2 in a genetically engineered mouse model of soft tissue sarcoma. Although GCN2 was both overexpressed and activated in the sarcomas, loss of GCN2 had no effect on tumor growth or animal survival. It appears that the tumors acquired various mechanisms to compensate for GCN2 loss, resulting in maintenance of ISR signaling. Sarcomas derived from GCN2^−/−^ C57BL6 mice specifically demonstrated high levels of PERK activation, while sarcomas derived from GCN2^−/−^ mixed background mice upregulated ATF4 even though eIF2α phosphorylation was reduced. Although these results do not address whether there is a direct role for PERK or ATF4 in compensating for GCN2 loss, we speculate that the development of these alternative signaling mechanisms indicates that the ISR is crucial for tumor cell survival. We also demonstrate that many ISR-related genes are overexpressed in human sarcoma samples, indicating our observations in the mouse model are clinically relevant.

These experiments highlight the importance of using genetically engineered mouse models of cancer to study the tumor microenvironment. We previously utilized flank xenograft models to study the role of GCN2 in tumorigenesis and found that Ras transformed GCN2^−/−^ MEFs formed extremely small tumors, while stable knockdown of GCN2 in the HT1080 human sarcoma cell line completely blocked tumor growth^16^. These results can most likely be attributed to the subcutaneous microenvironment of the xenograft model. When tumor cells are injected beneath the skin, they initially lack a blood supply to provide them with oxygen and nutrients. Loss of GCN2 renders cells extremely sensitive to amino acid deprivation^16^, and thus they probably undergo apoptosis before they can establish vasculature to deliver nutrients to the tumor site. In autochthonous tumor models, tumors are exposed to physiologically relevant levels of nutrients as they develop, which helps to support their initial growth.

Genetically engineered mouse models are not without their limitations, however. One caveat of this particular model is that GCN2 is absent from the very beginning of tumor formation. A more clinically relevant approach would be to block GCN2 activity after initial tumor development. In this type of model, GCN2 inhibition may slow tumor growth since the tumors have time to become reliant upon GCN2 to survive nutrient stress. This could be modeled genetically by utilizing dual recombinase technology^28^,^29^ to excise GCN2 after activation of Kras^G12D^ and deletion of p53 to initiate sarcomagenesis. Moreover, our results do not exclude a potential role of GCN2 in tumor growth in other settings or tissues of origin, such as epithelial tumors.

The ultimate goal of studying the roles of ISR kinases in mouse models is to determine the therapeutic efficacy of kinase inhibition on tumor growth. Several specific small molecule inhibitors of PERK have been identified^30^,^31^,^32^, while the currently known GCN2 inhibitors are non-specific and require further development^33^. Of these drugs, the PERK inhibitor GSK2656157 has been tested in xenograft models, with promising results^34^. However, our current work suggests that testing small molecule inhibitors of the ISR will yield more accurate results in GEMMs, rather than xenograft models. As the characterization of ISR inhibitors continues, GEMMs should become an important tool to evaluate their effects.

A second clinically relevant finding from this study is the mechanism by which tumors compensate for GCN2 loss, as tumors treated with small molecular inhibitors of ISR kinases may rely on similar pathways to become drug resistant. In this study, we observed PERK activation in the absence of GCN2. This suggests that combining inhibitors against multiple ISR kinases might effectively combat drug resistance. We also observed upregulation of ATF4 independently of eIF2α phosphorylation status. ATF4 is more difficult to directly target because it is a transcription factor. However, the downstream processes that ATF4 regulates, such as autophagy, amino acid biosynthesis, and redox signaling, are more amenable drug targets. Combination treatment with an ISR inhibitor and inhibitors of ATF4-regulated processes may also be an effective method to block signaling through this pathway. One ATF4-regulated process of particular interest is autophagy. We previously showed that loss of GCN2 compromises autophagy induction in response to glutamine deprivation in human fibrosarcoma cells^16^. While autophagy is difficult to measure *in vivo* due to its dynamic nature and lack of reliable markers, it would be interesting to determine if GCN2^−/−^ sarcomas have a reduced ability to undergo autophagy and if this deficiency could be therapeutically exploited in combination with GCN2 inhibition. Continued studies with genetically engineered mouse models will provide insights into useful therapies to target this pathway in cancer.

## Methods

### Ethics statement

All animal experiments were performed in accordance with the “Guide for the Care and Use of Laboratory Animals” of the National Research Council of the National Academies. All animal experiments were approved by the University of Pennsylvania Institutional Animal Care and Use Committee. Use of recombinant virus in animals was approved by the University of Pennsylvania Institutional Biosafety Committee.

### Mice and soft tissue sarcoma generation

GCN2^−/−^ mice (Jackson Laboratories), LSL-Kras^G12D/wt^ mice, and p53^fl/fl^ mice were previously described^3^,^35^,^36^. LSL-Kras^G12D/wt^;p53^fl/fl^ mice were injected with 2.5 × 10^7^ PFU of Ad5CMVcre (University of Iowa Gene Transfer Vector Core) in the upper leg muscle to induce sarcomas. Adenovirus was prepared by diluting in MEM media, adding CaCl_2_ to a final concentration of 9.6 mM, and incubating for 15 minutes to allow calcium phosphate precipitates to form. Animals were monitored three times weekly for tumor formation by palpitation. Once detected, tumors were measured three times weekly by calipers. The tumor volume was calculated for each individual mouse as [(tumor leg length x tumor leg width^2^ × 0.52) – (normal leg length x normal leg width^2^ × 0.52)]. Mice were euthanized once the tumor volume met or exceeded 1000 mm^3^. Tumor tissue and normal muscle tissue were harvested from mice and either snap-frozen in liquid nitrogen for western blot and qPCR analysis or fixed overnight in 10% neutral buffered formalin for histopathological analysis.

### Histopathology

Tumors and normal muscle tissue were rinsed in PBS and fixed in 10% neutral buffered formalin overnight. Fixed tissues were washed with a series of PBS, 50% ethanol, 70% ethanol, 95% ethanol, and 100% ethanol for one hour each. Tissues were then embedded in paraffin, sectioned, and stained with hematoxylin and eosin.

### Immunohistochemistry

Paraffin embedded tissues were dewaxed with a series of xylene, 100% ethanol, 95% ethanol, 80% ethanol, 70% ethanol, and distilled water washes. Antigen retrieval was carried out by incubating slides for 10 minutes at 95 °C in universal antigen retrieval solution (R&D Systems). Endogenous peroxidases were quenched with a 15 minute incubation in 2.25% hydrogen peroxide. Endogenous avidin and biotin were blocked using an avidin/biotin blocking kit (Vector Laboratories). Tissue sections were blocked using normal goat serum, and then incubated in CA9 antibody (Millipore) diluted 1:50 overnight at 4 °C. Sections were washed twice with PBS for five minutes each. Tissue sections were then incubated in biotinylated rabbit IgG (Vector Laboratories) for 30 minutes at 37 °C. Sections were washed twice in PBS for five minutes each. Slides were incubated in Vectastain Elite ABC solution (Vector Laboratories) for 30 minutes at 37 °C, and then washed twice in PBS for five minutes each. Signal was developed using 3,3’-diaminobenzidine (DAB) peroxidase substrate (Vector Laboratories). Slides were washed in water and counterstained with hematoxylin. Tissues were dehydrated with a series of water, 70% ethanol, 95% ethanol, 100% ethanol, and xylene washes and then mounted.

### Western blotting

50 mg pieces were cut from snap-frozen tumors or normal muscle and placed on ice in tissue lysis buffer (50 mM HEPES, pH 7.4; 150 mM NaCl, 1.5 mM MgCl_2_, 1 mM EGTA, 1% Triton X-100, 1 mM phenylmethylsulfonyl fluoride, 1X Complete Mini protease inhibitor cocktail [Roche], and 1X phosphatase inhibitor cocktail 2 [Sigma]). Tissues were homogenized on ice by electric homogenizer and then incubated on ice for 10 minutes. Lysates were cleared by centrifugation at maximum speed for 10 minutes. Protein concentrations of lysates were determined using 660 nm Protein Assay Reagent (Thermo Scientific), according to the manufacturer’s protocol. Equal amounts of protein were resolved on 4–15% gradient Tris-HCl polyacrylamide gels and transferred to polyvinylidene fluoride membranes. Membranes were incubated in blocking buffer (20 mM Tris, 137 mM NaCl, 5% non-fat dried milk, 0.1% Tween-20; pH 7.6), and then incubated in primary antibody diluted in washing buffer (20 mM Tris, 137 mM NaCl, 1% non-fat dried milk, 0.1% Tween-20; pH 7.6). After primary antibody incubation, membranes were washed three times with washing buffer, incubated with secondary antibody diluted in washing buffer, and washed three more times with washing buffer. Proteins were visualized by incubating membranes in ECL chemicals and exposing to film. Bands were quantified using ImageJ software. The following primary antibodies were used: β-tubulin, eIF2α, GCN2, p-eIF2α, PERK, p-PERK (all from Cell Signaling), ATF4, PKR (both from Santa Cruz), p-GCN2 (Bioss), and p-PKR (Millipore). The secondary antibodies used were anti-rabbit HRP and anti-mouse HRP from Thermo Scientific.

### Copy number analysis

Genomic DNA was isolated from snap frozen tumor or normal muscle tissue using a DNeasy Blood and Tissue Kit (QIAgen). DNA was digested with RNase A to remove any contaminating RNA. Copy number analysis on genomic DNA was performed using a TaqMan copy number assay with primers recognizing exon three of mouse ATF4 (Applied Biosystems, assay ID Mm00618542_cn), according to the manufacturer’s protocol. The ATF4 copy number assay was duplexed with a TaqMan copy number reference assay against TRFC (Applied Biosystems) for normalization. Data were analyzed using Copy Caller software.

### qPCR

RNA was isolated from homogenized snap frozen tumor tissue using an RNeasy Mini Kit (QIAgen). RNA was reversed transcribed into cDNA using random hexamer primers and AMV reverse transcriptase (Promega). qPCR was performed on the resulting cDNA templates using Power SYBR Green Master Mix (Applied Biosciences). qPCR reaction conditions were 50 °C for two minutes, 95 °C for 10 min, followed by 40 cycles of 95 °C for 15 seconds and 60 °C for one minute. Data were analyzed using QuantStudio Real-Time PCR Software. A list of primers used is available in Supplementary Table S1.

### ATF4 sequencing

Genomic DNA was isolated from tumors as described under “copy number analysis.” Exons 1, 2, and 3 of ATF4 were individually amplified by PCR using Herculase II high fidelity DNA polymerase (Agilent Technologies). PCR reaction conditions were 95 °C for two minutes; 30 cycles of 95 °C for 20 seconds, 55 °C for 20 seconds, and 72 °C for 30 seconds; and 72 °C for three minutes. A list primers used is available in Supplementary Table S2. PCR products were purified using a QIAquick PCR Purification kit (QIAgen). Purified PCR products were sequenced at the University of Pennsylvania Genomics Analysis Core.

### Statistical analysis

Survival data were analyzed using the log-rank (Mantel-Cox) test. In Oncomine, differential expression analysis is performed using an independent, two-sample, one-tailed Welch’s t-test. All other data were analyzed using a two-tailed Welch’s t-test. A p-value less than 0.05 was considered statistically significant for all analyses.

## Additional Information

**How to cite this article**: Lehman, S. L. *et al*. Signaling through alternative Integrated Stress Response pathways compensates for GCN2 loss in a mouse model of soft tissue sarcoma. *Sci. Rep*. **5**, 11781; doi: 10.1038/srep11781 (2015).

## Supplementary Material

Supplementary Information
