# Supplementary material for: Signaling through alternative Integrated Stress Response pathways compensates for GCN2 loss in a mouse model of soft tissue sarcoma
Source: Sci Rep. 2015 Jun 30;5:11781. doi: 10.1038/srep11781 (PMC4485314; doi:10.1038/srep11781)
Supplement: Supplementary Information [file srep11781-s1.pdf]

Signaling through alternative Integrated Stress Response pathways compensates for GCN2 loss in a mouse model of soft tissue sarcoma

Stacey L. Lehman[1], Sandra Ryeom[2], Constantinos Koumenis[1*]

[1]Deparment of Radiation Oncology, Perelman School of Medicine at the University of Pennsylvania, Philadelphia, PA, USA 19104

[2]Department of Cancer Biology, Perelman School of Medicine at the University of Pennsylvania, Philadelphia, PA, USA 19104

*Corresponding author: costas.koumenis@uphs.upenn.edu

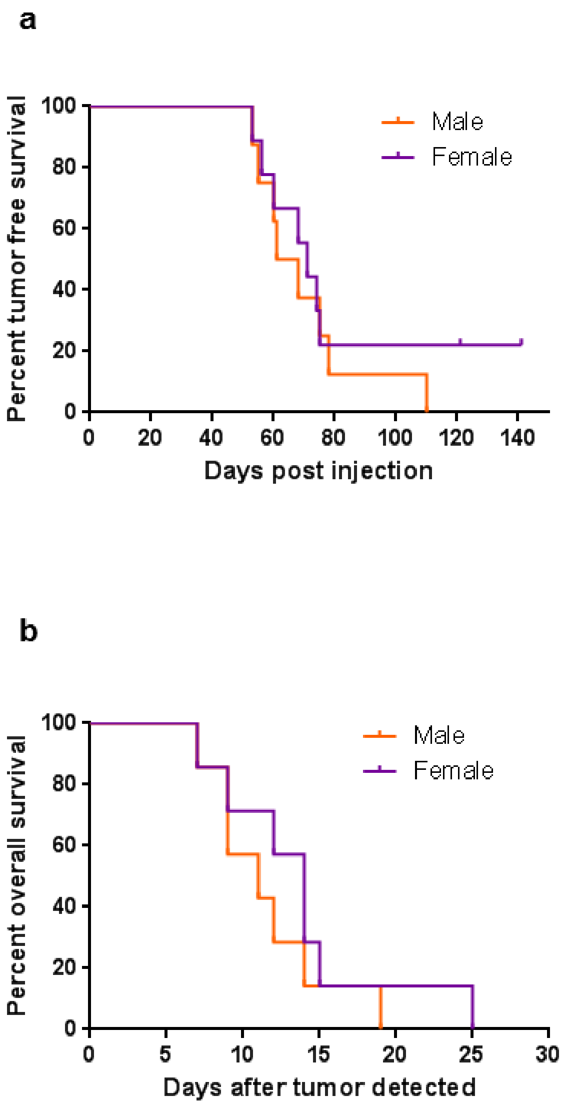

Supplementary Figure S1. Sex does not affect survival of sarcoma-bearing mixed background mice.

Supplementary Figure S1. Sex does not affect survival of sarcoma-bearing mixed background mice.

a. Kaplan-Meier curves depicting the time from Ad-cre injection to tumor formation for males and females. Results were not statistically significant.

b. Kaplan-Meier curves depicting the time from tumor detection to euthanasia for males and females. The major euthanasia criterion was a tumor volume exceeding 1000 mm^3. Results were not statistically significant.

Supplementary Information: ***Lehman, et. al. Signaling through alternative Integrated Stress Response pathways compensates for GCN2 loss in a mouse model of soft tissue sarcoma***

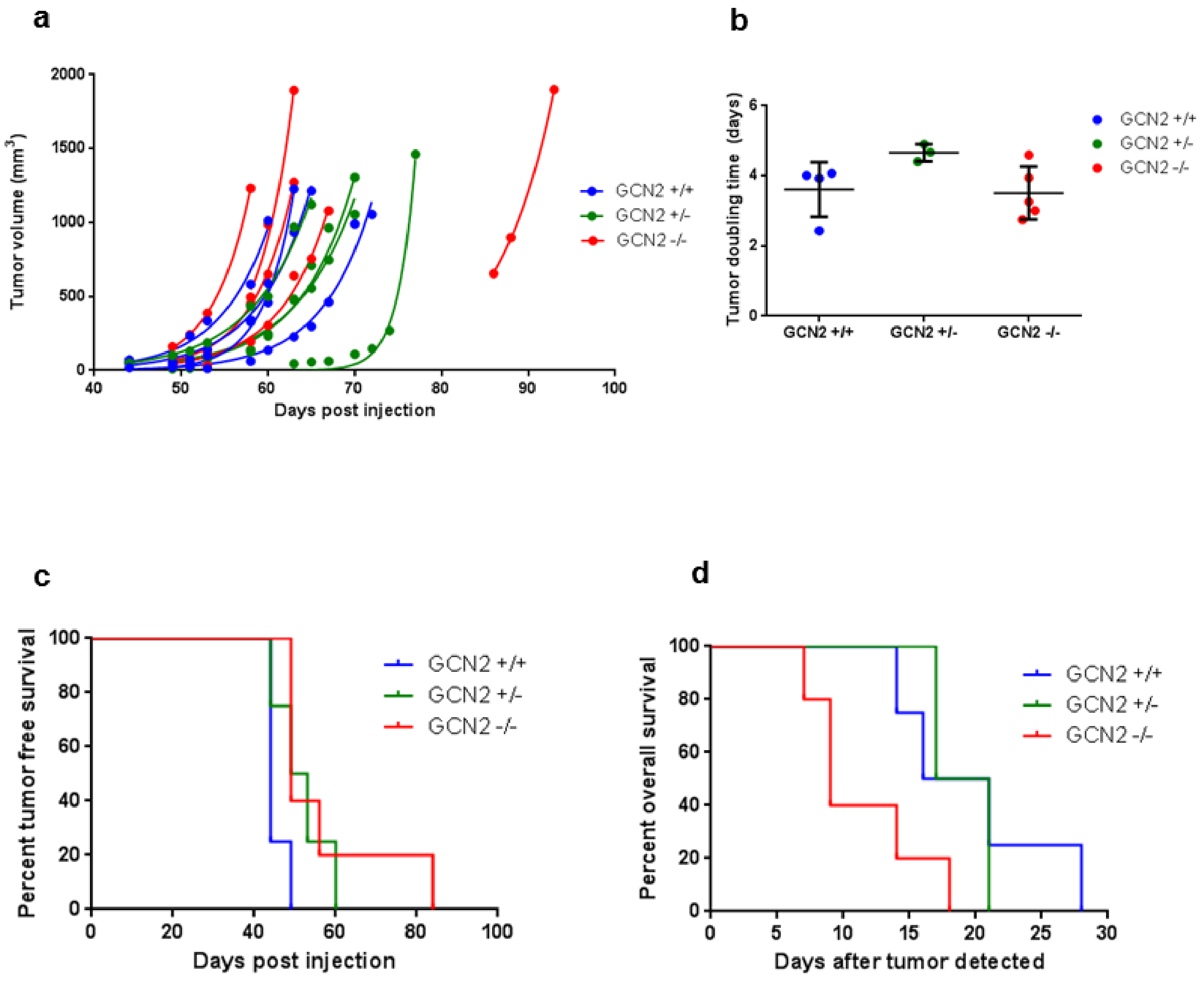

Supplementary Figure S2. GCN2 does not affect tumor growth or survival of sarcoma-bearing mice on a C57BL6 background

Supplementary Figure S2. GCN2 does not affect tumor growth or survival of sarcoma-bearing mice on a C57BL6 background.

a. Measurements of tumor volume over time in GCN2$^{+/+}$, GCN2$^{+/-}$, and GCN2$^{-/-}$ C57BL6 mice. The line through the set of measurements for each mouse represents the best fit exponential growth equation of the tumor.
b. Tumor doubling time was calculated from the best fit exponential growth equations shown in (a). The average doubling time ± standard deviation is depicted for each genotype. Results were not statistically significant.
c. Kaplan-Meier curves depicting the time from Ad-cre injection to tumor formation for each GCN2 genotype. Results were not statistically significant.
d. Kaplan-Meier curves depicting the time from tumor detection to euthanasia for each GCN2 genotype. The major euthanasia criterion was a tumor volume exceeding 1000 mm^3. Results were not statistically significant.

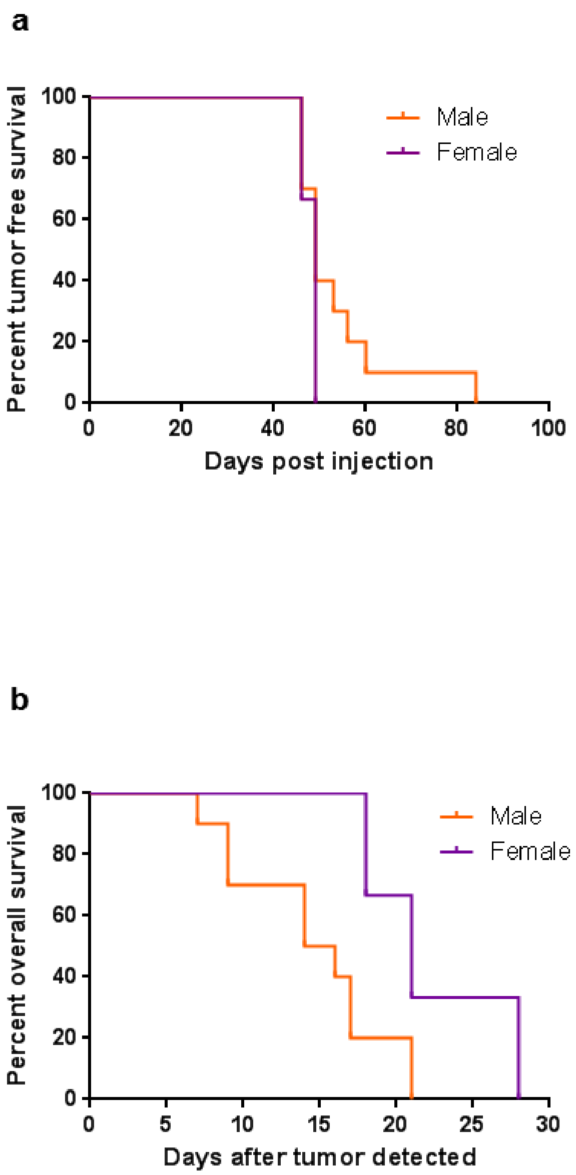

Supplementary Figure S3. Sex does not affect survival of sarcoma-bearing C57BL6 mice.

Supplementary Figure S3. Sex does not affect survival of sarcoma-bearing C57BL6 mice.

a. Kaplan-Meier curves depicting the time from Ad-cre injection to tumor formation for males and females. Results were not statistically significant.

b. Kaplan-Meier curves depicting the time from tumor detection to euthanasia for males and females. The major euthanasia criterion was a tumor volume exceeding 1000 mm^3. Results were not statistically significant.

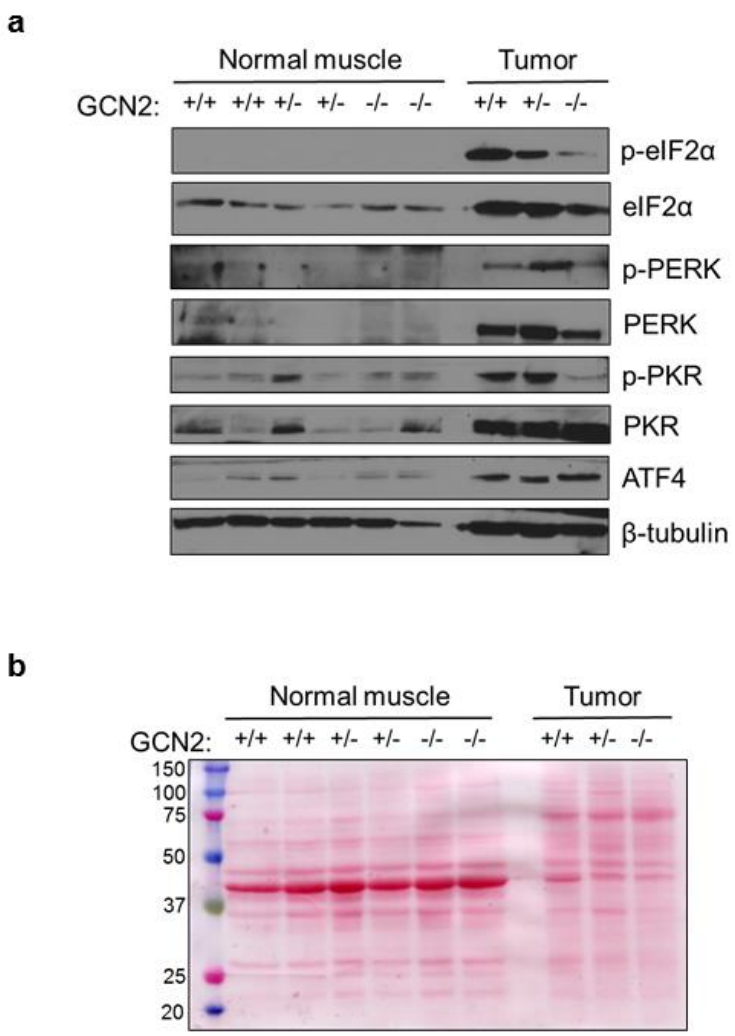

Supplementary Figure S4. ISR protein are overexpressed and activated in sarcomas as compared to normal muscle tissue.

Supplementary Figure S4. ISR proteins are overexpressed and activated in sarcomas.

a. Western blot analysis for various ISR proteins and their phosphorylated, active forms in both normal muscle tissue and tumor tissue. β-tubulin was used as a loading control.

b. Ponceau staining of total tissue lysates from normal muscle and tumor tissue.

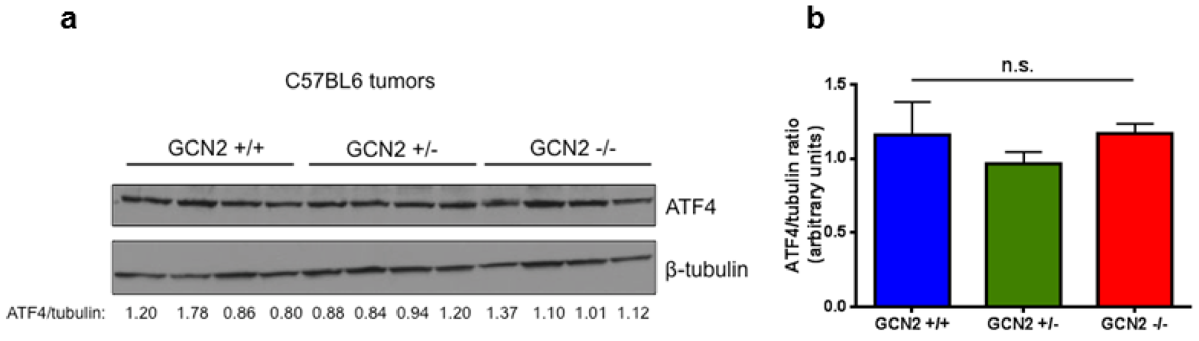

Supplementary Figure S5. Loss of GCN2 does not affect ATF4 levels in C57BL6 sarcomas

Supplementary Figure S5. Loss of GCN2 does not affect ATF4 levels in C57BL6 sarcomas.

c. Western blot analysis on tumor homogenates for levels of ATF4 in C57BL6 tumors. β-tubulin was used as a loading control. The ratio of ATF4 to β-tubulin is displayed below each blot. ATF4 was probed on the same blot as total and phospho-PERK and total and phospho-PKR (Figure 4c), so the β-tubulin loading control is the same in both figures.

d. Graphical representation of the ATF4 to β-tubulin ratios calculated from the blots in (a). Data are represented as the average value for each genotype ± standard error of the mean. Results are not statistically significant.

SNP profile A: NM_009716.3

```
   1 ggctaggtgt cccaccccgc cttgtaagac accggaaatt cgtcaacgag cgatccctcc
  61 gcgctccgga accagaccgc ggctggtcgt caacctataa agtagtgcac ttttctctca
 121 tggggccttt aggacgatct ctaacgccac agttaccccc cgagcacagc ggagaagggt
 181 tggggcgggc gagggcgtca gtgagaggcg gtttggtggc gctgcggtag gatcacgtga
 241 ccacagtggg aggatacgcg gtgtgctgcg tccctggccg aggctataaA gggcgggttt
 301 agggcgtgcc gccgccattt ctgcttgctg tctgccggtt tAagttgtgT gctcgggtgt
 361 ccctttcctc ttcccctccc gcagggcttg cggccaccat ggcgtattag aggcagcagt
 421 gcctgcggca gcgttggcct ttgcagcggc ggcagcagca ccaggctctg cagcggcaac
 481 ccccaccggc ctaagccatg gcgctcttca cgaaatccag cagcagtgtt gctgtaacgg
 541 acaaagatac cttcgagtta agcacattcc tCgaatccag caaagcccca caacatgacc
 601 gagatgagct tcctgaacag cgaagtgttg gcgggggact tgatgtcccc cttcgaccag
 661 tcgggtttgg gggctgaaga aagcctaggt ctcttagatg actatctgga ggtggccaag
 721 cacttgaaac ctcatgggtt ctccagcgac aaggcgggct cctcggaatg gccggctatg
 781 gatgatggct tggccagtgc ctcagacacc ggcaaggagg atgccttttc cgggacagat
 841 tggatgttgg agaaaatgga tctgaaagag tttgacttcg atgctctgtt tcgaatggat
 901 gacctggaaa ccatgccaga tgagctcttg accacgttgg atgacacatg tgatcttttt
 961 gcccctctag tccaagagac taataaggag ccccctcaga cagtgaaccc aattggccat
1021 ctcccagaaa gtttaataaa agtcgaccag gttgccccct ttacattctt gcagcctttc
1081 ccctgttccc caggggttct gtcttccact ccagagcatt cctttagttt agagctaggc
1141 agtgaagttg atatctctga aggagacagg aagcctgact ctgctgctta cattactcta
1201 atccctccat gtgtaaagga ggaagacact ccctctgaca atgacagtgg catctgtatg
1261 agcccAgagt cctacctggg ctctccccag catagcccct ccacctccag ggccccacca
1321 gacaatctgc cttctccagg tggttcccgt gggtctcctc ggcccaaacc ttatgaccca
1381 cctggagtta gtttgacagc taaagtgaag actgagaaat tggataagaa gctgaaaaag
1441 atggagcaaa acaagacagc agccactagg taccgccaga agaagcgggc tgagcaggag
1501 gccctcactg gcgagtgtaa ggagctagaa aaaaagaatg aggctctgaa agagaaggca
1561 gattctctgg ccaaggagat ccagtatctg aaagacctga tagaagaggt ccgtaaggca
1621 agggggaaga agagagttcc gtaatagggt agtcaggtgc tttgtgcttg tacatagtct
1681 tgtgttgctg tgtttgctgt aataaattat tttgtagtga aagtacctaa aaaaaaaaaa
1741 aaaaaa
```

SNP profile B: NM_009716.3

```
   1 ggctaggtgt cccaccccgc cttgtaagac accggaaatt cgtcaacgag cgatccctcc
  61 gcgctccgga accagaccgc ggctggtcgt caacctataa agtagtgcac ttttctctca
 121 tggggccttt aggacgatct ctaacgccac agttaccccc cgagcacagc ggagaagggt
 181 tggggcgggc gagggcgtca gtgagaggcg gtttggtggc gctgcggtag gatcacgtga
 241 ccacagtggg aggatacgcg gtgtgctgcg tccctggccg aggctataaG gggcgggttt
 301 agggcgtgcc gccgccattt ctgcttgctg tctgccggtt tGagttgtgC gctcgggtgt
 361 ccctttcctc ttcccctccc gcagggcttg cggccaccat ggcgtattag aggcagcagt
 421 gcctgcggca gcgttggcct ttgcagcggc ggcagcagca ccaggctctg cagcggcaac
 481 ccccaccggc ctaagccatg gcgctcttca cgaaatccag cagcagtgtt gctgtaacgg
 541 acaaagatac cttcgagtta agcacattcc tGgaatccag caaagcccca caacatgacc
 601 gagatgagct tcctgaacag cgaagtgttg gcgggggact tgatgtcccc cttcgaccag
 661 tcgggtttgg gggctgaaga aagcctaggt ctcttagatg actatctgga ggtggccaag
 721 cacttgaaac ctcatgggtt ctccagcgac aaggcgggct cctcggaatg gccggctatg
 781 gatgatggct tggccagtgc ctcagacacc ggcaaggagg atgccttttc cgggacagat
 841 tggatgttgg agaaaatgga tctgaaagag tttgacttcg atgctctgtt tcgaatggat
 901 gacctggaaa ccatgccaga tgagctcttg accacgttgg atgacacatg tgatcttttt
 961 gcccctctag tccaagagac taataaggag ccccctcaga cagtgaaccc aattggccat
1021 ctcccagaaa gtttaataaa agtcgaccag gttgccccct ttacattctt gcagcctttc
1081 ccctgttccc caggggttct gtcttccact ccagagcatt cctttagttt agagctaggc
1141 agtgaagttg atatctctga aggagacagg aagcctgact ctgctgctta cattactcta
1201 atccctccat gtgtaaagga ggaagacact ccctctgaca atgacagtgg catctgtatg
1261 agcccGgagt cctacctggg ctctccccag catagcccct ccacctccag ggccccacca
1321 gacaatctgc cttctccagg tggttcccgt gggtctcctc ggcccaaacc ttatgaccca
1381 cctggagtta gtttgacagc taaagtgaag actgagaaat tggataagaa gctgaaaaag
1441 atggagcaaa acaagacagc agccactagg taccgccaga agaagcgggc tgagcaggag
1501 gccctcactg gcgagtgtaa ggagctagaa aaaaagaatg aggctctgaa agagaaggca
1561 gattctctgg ccaaggagat ccagtatctg aaagacctga tagaagaggt ccgtaaggca
1621 agggggaaga agagagttcc gtaatagggt agtcaggtgc tttgtgcttg tacatagtct
1681 tgtgttgctg tgtttgctgt aataaattat tttgtagtga aagtacctaa aaaaaaaaaa
1741 aaaaaa
```

Supplementary Figure S6. Mixed background sarcomas expressed *ATF4* mRNA with two distinct patterns of SNPs.

Supplementary Figure S6. Mixed background sarcomas express *ATF4* mRNA with two distinct patterns of SNPs

Full sequence of the *ATF4* mRNAs encoded by each of the SNP profiles identified in mixed background sarcomas. The SNPs are in capital letters and highlighted in blue. The start and stop codons of uORF1, uORF2, and the ATF4 ORF are highlighted in green, pink, and yellow, respectively.

Supplementary Table S1. Primer sequences used for qPCR analysis.

Primer Name	Sequence (5' to 3')
18S forward	CAATTACAGGGCCTCGAAAG
18S reverse	AAACGGCTACCACATCCAAG
Asns forward	GCAGTGTCTGAGTGCGATGAA
Asns reverse	TCTTATCGGCTGCATTCCAAAC
Atf3 forward	GAGGATTTTGCTAACCTGACACC
Atf3 reverse	TTGACGGTAACTGACTCCAGC
Atf4 forward	CCTGAACAGCGAAGTGTTGG
Atf4 reverse	TGGAGAACCCATGAGGTTTCAA
β-actin forward	GGCTGTATTCCCCTCCATCG
β-actin reverse	CCAGTTGGTAACAATGCCATGT
β-tubulin forward	GATCGGTGCTAAGTTCTGGGA
β-tubulin reverse	AGGGACATACTTGCCACCTGT
GCN2 forward	CCCGGACATACTCCTCAGGAA
GCN2 reverse	GGCTACCCACAGAGAAATGGA
Ulk1 forward	ACATCCGAGTCAAGATTGCTG
Ulk1 reverse	GCTGGGACATAATGACCTCAGG
XBP1 spliced forward	CTGAGTCCGCAGCAGGTG
XBP1 spliced reverse	TCCAGAATGCCCAAAAGG

Supplementary Table S2. Primer sequences used to amplify ATF4 for sequencing

Primer Name	Sequence (5' to 3')
ATF4 exon 1 forward	GTGATAACCTGGCAGCTTCG
ATF4 exon 1 reverse	CAGGCAACGCAAACAAGG
ATF4 exon 2 forward	TCCAGGCTCTTCACGAAATC
ATF4 exon 2 reverse	CCATATGAAAAGGGCACACC
ATF4 exon 3 forward	TTCTGCCTCCCGAATATGAC
ATF4 exon 3 reverse	CAAGCACAAAGCACCTGACT
